# From fossils to genomes: decoding the insect world

**DOI:** 10.1093/nsr/nwaf055

**Published:** 2025-02-22

**Authors:** Jun Xu, Wei Zhang

**Affiliations:** State Key Laboratory of Plant Trait Design, Center for Excellence in Molecular Plant Sciences, Chinese Academy of Sciences, China; State Key Laboratory of Gene Function and Modulation Research, School of Life Sciences, Peking University, China

Insect diversity constitutes one of the most remarkable biological phenomena on our planet, characterized by intricate and multifaceted relationships with human society. Functioning as both pollinators and decomposers, insects serve as indispensable components in sustaining ecological equilibrium. This biodiversity is quantitatively reflected in the existence of over one million recorded species [1], while qualitatively manifested through diverse human-insect interactions, encompassing both detrimental disease vectors and beneficial species that enhance agricultural productivity. The ecological significance of insects extends far beyond conventional perceptions, as they facilitate nutrient cycling, regulate pest populations, provide essential food resources for wildlife, and inspire biomimetic innovations in science and technology. Consequently, the comprehension and conservation of insect diversity have become imperatives for maintaining ecosystem stability and safeguarding human well-being.

Insects are far more than just insects; *Drosophila melanogaster* has served as a model organism for over a century, contributing significantly to our understanding of numerous fundamental biological questions. Many genetic tools have been developed in *Drosophila* for the study of fundamental biological questions in mammals [2]. In addition to the well-established model organism *D. melanogaster*, the extraordinary diversity within the insect world offers an invaluable genetic repository that facilitates a wide range of scientific investigations. Butterflies serve as an ideal system for investigating evolutionary and ecological questions such as migration patterns and mimicry phenomena, while the flight stabilization mechanisms of dragonflies have inspired solutions to multiple engineering challenges in helicopter design. Additionally, with respect to resource insects, the silk proteins produced by domestic silkworms constitute a valuable source for silk manufacturing and other industrial applications.

In this Special Topic, *National Science Review* showcases cutting-edge developments in insect (entomology) research, including one perspective and five original research articles. In the perspective, Xu *et al.* reviewed the capacity of black soldier fly (BSF) larvae to degrade various classes of antibiotics, along with the underlying mechanisms of biodegradation. Remediation of antibiotic pollutants from organic wastes by the larvae is promising not only due to their exceptional degradation capacity, but also their ability to assimilate residual nutrients from the wastes into their biomass, which can be used as animal feed, biodiesel and chitin. Despite this potential, the challenges of the field such as limited understanding of the toxicity of the degradation intermediates and the limited degradation efficacy for certain antibiotics remain to be tackled. To advance the application of the technology, Xu *et al.* proposed that future research should focus on identifying key microbial and insect genes involved in the degradation and characterizing the chemical biodegradation pathway during the process [3].

In the first research article, Vršanský *et al.* investigated exceptional Jurassic scale insect fossils preserved in Lebanese amber through the application of microscopic and mass spectrometry techniques analysis. This groundbreaking research not only furnishes pivotal evidence for understanding insect evolution but also contributes substantially to our comprehension of Jurassic ecosystem dynamics [4]. This discovery has established a new field within the study of Jurassic amber fossils.

Two research articles focus on dissecting insect genomes to provide evidence for comprehending the mechanisms of adaptive evolution in biological systems. Peng *et al.* reported that the population structure of the *Chilo suppressalis* comprises five distinct populations in China, each exhibiting significant genetic differences. The study reveals that *C. suppressalis* harbors a diverse array of adaptive variations, which facilitate its adaptation to varying environments. This natural genetic diversity serves as a valuable resource for investigating environmentally driven adaptive evolution. Innovatively, it is proposed that the risk of pest outbreaks should be monitored through genetic means, and the expression of relevant genes or pathways should be further disrupted using green pesticides, thereby achieving a strategy for green pest control [5]. Huang *et al.*, provided genomic-level insights for the absence of eyes and wings, along with the evolution of extended legs specialized for thriving within bats’ dense fur in bat flies. By employing small RNA sequencing techniques, they successfully detected an array of viruses, including both recognized and novel classifications, within bat flies. This study not only contributes novel insights on the evolutionary processes governing bat fly parasitism but also introduces an innovative experimental platform for examining the mechanisms underlying the cross-species transmission of bat-associated viruses and the reciprocal evolutionary dynamics between bats and their viral pathogens [6].

Another two research articles investigated insect developmental and behavioral mechanisms through functional genomic and genetic manipulation approaches. Zhu *et al.* established a multi-organelle regulatory axis for juvenile hormone (JH) production and female reproduction in the American cockroach. Their studies bridged extracellular cues to intracellular signaling, it redefines endocrine regulation and offers novel targets for disrupting pest reproduction through JH pathway modulation [7]. Zhang *et al.* found that *Bactrocera dorsalis* males consume methyl eugenol (ME) and metabolize it into trans-coniferyl alcohol, which increases their leks’ attractiveness to females and enhances female mate selection. The ability to locate ME, crucial for this process, is controlled by a specific olfactory receptor neuron. This research reveals how plant volatiles, which are central to insect behavior-manipulation strategies like the male annihilation technique (MAT), influence insect mating behavior and provide a foundation for developing pest control methods targeting olfactory pathways [8].

To summarize, entomology is both an ancient discipline and one with unique allure. The presented articles in this special topic demonstrate cutting-edge developments and current directions in entomological research. In the future, entomology will increasingly reflect interdisciplinary integration, with emerging technologies significantly propelling its progress. In addition to genomic and gene-editing technologies, future advancements in artificial intelligence, big data analytics, and chemical biology etc. will significantly contribute to the progression of entomological studies. Furthermore, the advancement of entomology will reciprocally stimulate progress across various fields of the biological sciences.

We hope this Special Topic will enhance readers’ understanding of the significance of entomological research and provide new impetus for advancements in life science. We would like to express our gratitude to Prof. Jiatang Li, Prof. Jie Cui and Prof. Shizhong Xu for their assistance in coordinating the review process, as well as to the reviewers for their insightful comments and suggestions. Additionally, we extend our sincere appreciation to the editorial staff of *National Science Review* for their unwavering support.
